# Identification of Two Exosomal miRNAs in Circulating Blood of Cancer Patients by Using Integrative Transcriptome and Network Analysis

**DOI:** 10.3390/ncrna8030033

**Published:** 2022-05-12

**Authors:** Andrés Rincón-Riveros, Josefa Antonia Rodríguez, Victoria E. Villegas, Liliana López-Kleine

**Affiliations:** 1 Bioinformatics and Systems Biology Group, Universidad Nacional de Colombia, Bogotá 111221, Colombia; warinconr@unal.edu.co; 2 Cancer Biology Research Group, Instituto Nacional de Cancerología, Bogotá 111221, Colombia; jrodriguez@cancer.gov.co; 3 Centro de Investigaciones en Microbiología y Biotecnología-UR (CIMBIUR), Facultad de Ciencias Naturales, Universidad del Rosario, Bogotá 111221, Colombia; 4 Department of Statistics, Faculty of Science, Universidad Nacional de Colombia, Bogotá 111221, Colombia

**Keywords:** miRNA, extracellular vesicles, cancer

## Abstract

Exosomes carry molecules of great biological and clinical interest, such as miRNAs. The contents of exosomes vary between healthy controls and cancer patients. Therefore, miRNAs and other molecules transported in exosomes are considered a potential source of diagnostic and prognostic biomarkers in cancer. Many miRNAs have been detected in recent years. Consequently, a substantial amount of miRNA-related data comparing patients and healthy individuals is available, which contributes to a better understanding of the initiation, development, malignancy, and metastasis of cancer using non-invasive sampling procedures. However, a re-analysis of available ncRNA data is rare. This study used available data about miRNAs in exosomes comparing healthy individuals and cancer patients to identify possible global changes related to the presence of cancer. A robust transcriptomic analysis identified two common miRNAs (miR-495-3p and miR-543) deregulated in five cancer datasets. They had already been implicated in different cancers but not reported in exosomes circulating in blood. The study also examined their target genes and the implications of these genes for functional processes.

## 1. Introduction

In the past three decades, the accumulation of gene expression data in databases al-lowed an extensive study to understand the molecular processes involved in diseases and their diagnosis using transcriptomic biomarkers. It also helped refine treatments and identified pharmacologically interesting genes [1]. High-throughput sequencing has recently allowed identifying and quantifying small RNAs (sRNAs) in eukaryotes, revealing their central role in diseases and their potential use as biomarkers. Since they can be found both in disease-affected tissue and extracellular vesicles circulating in the blood, they are far more useful for this purpose than transcriptomic gene biomarkers [2].

Extracellular vesicles are small structures with a lipid bilayer, produced by the endocytic machinery of the cell and secreted by most nucleated cells [3]. Exosomes and other types of extracellular vesicles circulating in the body fluids of cancer patients could be a source of diagnostic and prognostic cancer biomarkers. During the past decade, there was a dramatic increase in studies demonstrating that extracellular vesicles carry molecules of great biological and clinical interest. For example, exosomes contain non-coding RNAs, especially miRNAs, which have been strongly suggested to be associated with the development of cancer [4,5,6,7,8]. MicroRNAs are small non-coding RNAs of approximately 20–25 nucleotides (nt) in length, with the ability to modulate gene expression by binding to specific regions at the 3′ UTR end of genes, inducing the repression or complete degradation of mRNAs [9,10].

The first step towards a possible use of sRNAs (especially miRNAs) contained in exosomes is to explore available information related to their presence, relationship with disease, and potential use as biomarkers for prognosis, diagnosis, and treatment. Many sRNAs have been detected in recent years; consequently, a substantial amount of sRNA-related data comparing patients and healthy individuals is available at present, especially in cancer. Although there exists some specific knowledge on the role of miRNAs in isolated types of cancer, the use of these datasets for an overall and integrative analysis of vesicle content has been very limited. It can, however, provide significant insight about the potential use of non-coding RNAs contained in them and promote the use of liquid biopsies. Combining this information with evidence on the roles of sRNAs mainly in regulatory processes should deepen our understanding of the initiation, development, malignancy, and metastasis of cancer using noninvasive sampling procedures. This study partly fulfills this need.

Methods for analyzing gene expression data need to be adapted and integrated properly with information in the literature and databases to clarify and develop biological hypotheses about the roles of miRNAs in cancer [11,12]. In this study, we analyzed available data about miRNAs contained in exosomes comparing healthy individuals and cancer patients to identify possible global changes related to the presence of cancer. Using robust transcriptome analysis, we identified two common miRNAs (miR-495-3p and miR-543) deregulated in all five cancer datasets analyzed in this work. They had already been implicated in different cancer types but not reported in exosomes circulating in blood. These common miRNAs can serve as a starting point for monitoring tumorigenesis by studying their target genes and the involvement of these genes in functional processes. We, thus, investigated the molecular processes possibly affected by them by identifying their target genes using a network-based approach. We also discussed how the direction of deregulation in each cancer type and the molecular functions affected by their target genes make these two miRNAs potentially useful biomarkers in cancer.

## 2. Results

### 2.1. miRNA Expression in Different Types of Cancer

The established criteria for identifying miRNAs with differential expression in different types of cancer included a fold change >1 or ≤1 and a *p*-value adjusted by the FDR method <0.2. We chose this soft threshold given that our main goal was to globally identify potential central miRNAs common to several and very different types of cancer (Table 1) in the sense of a meta-analysis. Table S1 summarizes the results of the differential expression analysis.

To determine a possible miRNA master regulator between different tumor types, we inspected the differential expression datasets of these seven cancer types using a Venn diagram (Figure S1). In these datasets, we found that at least one miRNA with altered expression in common was found among five types of cancer. In gastric cancer and colorectal cancer, miR-495-3p and miR-543 were overexpressed, while in multiple myeloma, only miR-495-3p was overexpressed. In contrast, in prostate cancer, miR-495-3p and miR-543 were found to be deregulated; in glioblastoma, only miR-543 was deregulated (Table 2).

### 2.2. External Validation of Differentially Expressed miRNAs

We used the UALCAN tool [13] (http://ualcan.path.uab.edu/analysis.html, accessed on 20 March 2022) to test the expression of common miRNAs in the studied cancer types (except multiple myeloma, which is not available) (Table 2). Next, the findings were validated, including the direction of the deregulation of miRNAs present in extracellular vesicles.

Subsequently, a network was built with miRNAs in exosomes that overlapped among the five types of cancer in the miRNet web application (https://www.mirnet.ca/ accessed on 20 March 2022), to search for the target genes of these miRNAs.

### 2.3. miRNA–mRNA Bipartite Interaction Network

The constructed miRNA–mRNA network had 1121 nodes and 1255 edges based on information from the literature in the miRNet database. For miR-495-3*p*, we found connections with respect to 981 genes; on the other hand, for miR-543, there were connections with respect to 183 genes. Notably, two exosomal miRNAs’ shared connections to 45 genes (Figure S2) were as follows: *HMGA2*, *MIEF1*, *FNDC3B*, *ZNF703*, *KLHL15*, *SIGLEC14*, *AMD1*, *BACH1*, *CLTC*, *DDX3X*, *NOTCH2*, *NRAS*, *UBL3*, *PTP4A1*, *STX7*, *MATR3*, *WDFY3*, *ARL6IP1*, *C16orf72*, *LIN7C*, *GPCPD1*, *FBXO11*, *ADAM22*, *TMEM33*, *COL12A1*, *ANKRD13C*, *YPEL5*, *ACSL3*, *ZBTB1*, *BTBD3*, *DYNC1LI2*, *EGLN1*, *SERPINE2*, *RPLP1*, *ARHGAP29*, *SMARCA5*, *GNAQ*, *ZNF281*, *IRGQ*, *KIF5C*, *CFL1*, *NAP1L1*, *F1*, *PGAP1*, and *GFPT1*.

Of these common target genes of miR-495-3p and miR-543, *NRAS*, *HMGA2*, and *EGLN1* present in KEGG cancer metabolic pathways stand out (*p*-value < 0.05) (Figure S3). The top three target genes with the highest degree and betweenness in the bipartite network were *AKT1*, *JUN*, and *GSK3B*, which were involved in the miR-495-3p community. Interestingly, these hubs were not present in the large community detected with the Louvain algorithm. However, *GSK3B* (Glycogen Synthase Kinase 3 Beta) acts as a negative regulator in the phosphorylation of key cancer-related genes, such as *APC*, *JUN*, and *CTNNB1/beta-catenin*. For its part, *AKT1* is a regulator of *GSK3B* [14,15].

### 2.4. Community Detection and Functional Enrichment Analysis

**Figure 1:**
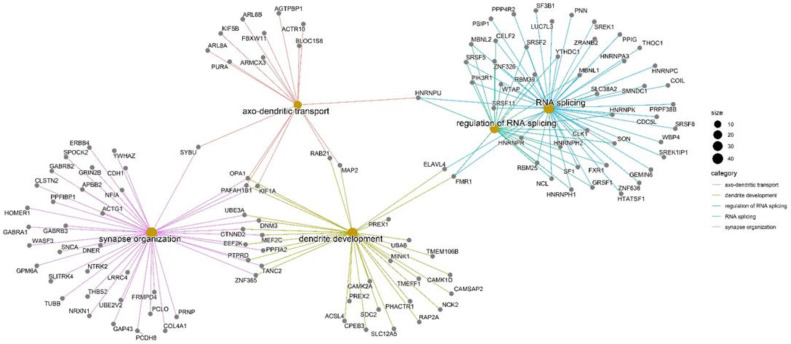
Enrichment network for results obtained from the hypergeometric enrichment test: linkages of genes, GO biological process, and enriched terms are connecting overlapping gene sets.

We used the Louvain algorithm to detect communities in the bipartite network. The community associated with miRNA-495-3p was the largest one, and it was further employed to perform functional enrichment analysis. For analysis, we selected overrepresented KEGG pathways and GO terms identified as significant based on Fisher’s exact test with a *p*-value adjusted by the Benjamini–Hochberg method of less than 0.05. Table 3 lists the top six metabolic pathways (Figure S4). The GO terms in the category of biological process (BP) with the highest representation among the genes of this community were synapse organization, axo-dendritic transport, RNA splicing, regulation of RNA splicing, and dendrite development. A network was built with the most representative terms to assess the principal biological process represented in miRNA–mRNA targets. It showed how these genes connect with other BPs of interest in transcriptomic and RNA regulation processes, which can be affected in diverse cancer types (Figure 1).

**Figure 2:**
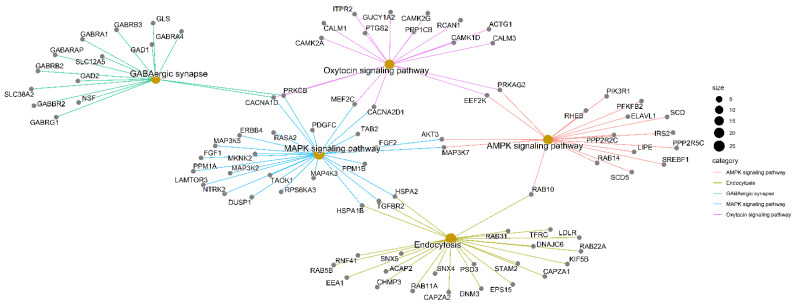
Gene-concept networks from the KEGG enrichment analysis; the beige circles represent pathways while the gray ovals indicate genes.

For the enrichment of KEGG metabolic pathways, we identified genes annotated to more than one metabolic pathway were also found, demonstrating the complexity of the biological processes. Among the top five metabolic pathways with a several members and low associated adjusted *p*-values, there were interconnections between potential target genes of miR-495-3*p* and ncRNA participating in several metabolic functions (Figure 2).

The community with the second largest number of members was the one associated with miRNA-543. This community had 70 members, on which functional enrichment was carried out with enrichKEGG from the clusterProfiler package. For this community, we only identified one metabolic pathway was identified with adjusted *p*-value < 0.05, namely, SNARE interactions in vesicular transport (SNAP23/STX4/STX7/STX1B). This pathway is composed of a family of proteins that participates in the biogenesis and secretion of extracellular vesicles, which has been studied in various fields such as autophagy and diseases such as Parkinson’s [16,17,18].

## 3. Discussion

Epigenetic modifications are closely associated with the development of cancer. Specifically, histone modifications, DNA methylation, and the regulation of gene expression by ncRNAs have been revealed to be related to this disease [19,20]. An miRNA can have hundreds or thousands of mRNAs targets; in turn, a single gene can be modulated by several miRNAs [21]. Therefore, it is necessary to bear in mind that the molecular signature of each type of tumor is unique. Cancer is considered a heterogeneous disease, due to which it is crucial to start focusing efforts on deciphering common patterns of different tumors [22,23].

Furthermore, miRNAs are closely related to other species of ncRNAs such as lncRNAs and circRNAs, generating a close network of interactions and competition for molecular targets. This network forms the basis for Salmena’s competitive endogenous RNA (ceRNA) hypothesis proposed in 2011 [24,25].

Given the complexity of molecular interactions, focusing only on a single type of biomolecule for the understanding of a complex disease such as cancer creates difficulties in the understanding of biological processes and disease development. Thus, it is vital to apply systems biology and network analysis, including protein–protein interaction networks, metabolites, and transcription factor genes [26,27,28]. Recent advances in systems biology and bioinformatics have also increased our understanding of heterogeneous interaction networks in cancer [29] and have highlighted the importance of miRNAs in cancer. Therefore, identifying two common miRNAs in five cancer types is an important starting point to develop biomarkers for diagnosis and treatment tuning.

Notably, we found that two miRNAs circulating in exosomes were upregulated in each cancer studied here in comparison to levels in healthy patients: hsa-miR-543 and hsa-miR-495-3*p*. External validation with TCGA data using the UALCAN tool highlighted that screening deregulated miRNAs in available databases generates valuable results. Although the literature has reported that hsa-miR-543 and hsa-miR-495-3*p* are involved in different types of cancer, there are no mentions of their detection in circulating exosomes as potential oncogenic markers. miR-543 has already been shown to suppress breast tumor cell viability, proliferation, and progression by repressing VCAN [30]. miR-543 also plays an oncogenic role in prostate cancer cells by suppressing Numb and promoting tumor growth, metastasis, and the acquisition of stem cell-like traits [31]. The knowledge that this miRNA is deregulated in different cancer types but is also related to other genes not directly involved in cancer renders this non-coding RNA a promising candidate for monitoring tumor-related processes; therefore, it should be a subject of further investigation [32]. CircTLK1 sequesters miR-495-3*p* by sponging, thus contributing to tumor growth and metastasis in renal cell carcinoma [33]. In contrast, the hypermethylation of the miR-495-3*p* promoter increases the expression of at least 10 epigenetically modified oncogenes that are overexpressed in gastric cancer [34]. Moreover, studies have demonstrated the tumor-suppressing potential of miR-495-3*p* in stomach, melanoma, and prostate tumors, which raises many questions about the role of this miRNA in all cancers analyzed here [35,36,37].

Our findings showed that miR-495-3*p* and miR-543, recognized modulators of carcinogenesis, were downregulated in prostate cancer. This result was possibly due to competition with other ncRNAs, such as NORAD and MCM3AP-AS1 lncRNAs that silence these miRNAs by increasing the expression of TRIP13 and the SLC39A10/PTEN/Akt axis, accelerating tumor progression [37,38]. We also found deregulated miR-543 in glioblastoma, which has been reported as a preoperative and classification biomarker in glioma [39]. In contrast, our results indicated that miR-495-3*p* and miR-543 are upregulated in gastric and colorectal cancer, in line with some published reports. In colorectal cancer, the overexpression of miR-543 was reported to increase chemoresistance by blocking tumor suppressor PTEN [40,41]. This miRNA also suppresses histone deacetylase SIRT1 in gastric tumor cells, increasing proliferation and tumor progression [42]. As for multiple myeloma, recent studies have indicated the importance of using non-invasive biomarkers to study this disease [43]. We found that miR-495-3*p* was deregulated, which has been proposed as a tumor suppressor in some hematological malignancies, such as acute myeloid leukemia and mixed lineage leukemia [44]. Previous studies suggested that miR495-3*p* modulates the expression of aquaporin-1 (AQP1), a protein with a potential role in osteosarcoma and multiple myeloma development [45,46,47].

To further investigate the role of these two common miRNAs, we identified their target genes using the miRNet tool. Experimental evidence allowed us to build a network where nodes were miRNAs and their target genes and edges represented interactions between them. Based on this network, network theory metrics were applied to find miRNA-mRNA regulatory patterns. The application of modularity algorithms for detecting communities allowed the identification of groups with a high density of connections. These groups of clustered genes associated with hsa-miR-543 and hsa-miR-495-3*p* indicated a closer community of relevant target genes, which helped clarify the potential global role of these target genes in cancer and physiological processes.

The metabolic pathways associated with the miRNA-495-3*p* community have already been described in cancer development. Gamma-aminobutyric acid (GABA) synapse is one of the main metabolic pathways of neurotransmitters in mammals, with implications in regulating inflammation and immune response. It has been found to promote cell proliferation due to the overexpression of the GABAA receptor activating MAPK signaling in brain, gastric, breast, and prostate cancers [48,49,50].

## 4. Material and Methods

**Figure 3:**
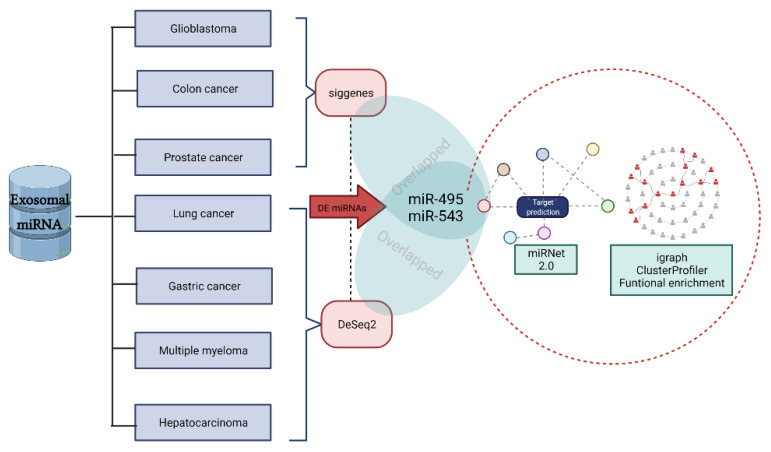
Flowchart of the pipeline employed for the multi-cancer exosomal miRNA study.

In this study, we reanalyzed openly available data on miRNAs contained in exosomes from recognized genomic databases. We examined datasets of cancer-associated exo-somes circulating in human plasma or serum, from tumor samples and normal controls, to detect differentially expressed miRNAs. We proceeded to predict the target genes of the most significant miRNAs, using an miRNA-gene bipartite network to illustrate the most important findings regarding the roles of miRNAs in biological functions and metabolic pathways (Figure 3).

### 4.1. High-Throughput Gene Expression Data Retrieval

For the present study, we retrieved datasets from the Gene Expression Omnibus (GEO, http://www.ncbi.nlm.nih.gov/geo, accessed on 1 June 2021), Expression Atlas (https://www.ebi.ac.uk/gxa, accessed on 5 June 2021), and Genomic Expression Archive (GEA, https://www.ddbj.nig.ac.jp/gea, accessed on 5 June 2021). We also applied specialized search tools, such as Google Dataset Search (https://datasesearch.reearch.google.com, accessed on 6 June 2021), OmicsDI (http://www.omicsdi.org, accessed on 6 June 2021), and DataMed (https://datamed.org, accessed on 7 June 2021), using the following keywords: “cancer”, “exosomes”, “RNAseq”, and “exosomal RNA”. Table 4 provides detailed information on the six RNAseq datasets selected for this study, with seven representative cancer types. All selected datasets had respective healthy or normal controls to perform differential expression analysis.

### 4.2. Data Processing

Each selected database was subject to quality control before processing, which involved filtering out miRNAs with low counts and outliers and independent normalization. To identify potentially deregulated miRNAs, we used the R DESeq2 and siggenes packages [55,56]. For this first filter of potentially deregulated miRNAs, we established a False Discovery Rate (FDR) of 20% and a fold-change threshold of >1.0.

### 4.3. Bipartite miRNA–Gene Network

Using miRNAs that passed the first filter in the five types of cancer studied to predict possible target genes, we applied the miRNet web tool (http://www.mirnet.ca/, accessed on 20 August 2021). In association with 20 databases, this tool forms a unique database of information on exosomes in various species [57]. The miRNA–mRNA interactions found in miRNet were processed with R package igraph [58]. Subsequently, we built a network with the miRNAs of exosomes overlapping among the five types of cancer in the miRNet web application, looking for the target genes of these miRNAs. As a source of the miRNAs, we selected “exosomes”, which is an advantage of miRNet and is achieved by linking ExoCarta to the analysis [59]. The versions used were miRTarBase v8.0 [60] and TarBase v8.0 [61] for gene search.

### 4.4. Network Community Detection

The basic topological properties of the network, such as degree (representing the level of connectedness), were analyzed using the R package igraph. For the detection of communities, we evaluated three algorithms, Louvain [62], label propagation [63], and Walktrap [64], in the same R package [58]. The communities found in the constructed interaction network were identified using the modularity index with respect to their members [65].

$$Q=\overset{k}{\underset{i=1}{\sum }}\left(e_{ii}-a_{i}^{2}\right)\;$$

### 4.5. Enrichment Analysis

We analyzed metabolic pathway overrepresentation (KEGG) and Gene Ontology (GO) using the enrichKEGG (KEGG metabolic pathways), enrichMKEGG (KEGG modules), and enrichGO functions of the R clusterProfiler package [66], after collecting terms and pathways with an adjusted *p*-value < 0.05, a minimum count of 10, and an enrichment factor > 1.5 (ratio between the observed counts and the counts expected by chance) for analysis.

## 5. Conclusions

Our study provides an integrative analysis of miRNA targets expressed in extracellular vesicles in different types of cancer, their possible relationships with vital physiological functions, and their potential roles in cancer. miRNAs and their relationships with target genes of diverse functional groups are valuable for diagnostic and therapeutic purposes. Beyond acting as potential transcriptomic biomarkers, they serve for monitoring disease status and treatment tuning because they are polyfunctional molecules: in certain types of tumors, they can play an oncogenic role (oncomiR) while acting as tumor suppressors in others. Therefore, there is much interest in further investigating the content of vesicles, including more data sets, which has not happened very often, and wet lab experiments. The use of miRNAs as biomarkers of cancer onset or progression and in monitoring therapeutic outcomes is promising. Nevertheless, considering their multiple roles and partners, their use needs to be preceded by large-scale prospective studies on various types of cancer to identify the molecular signatures of each disease.

## Figures and Tables

**Table 1:** Results summary of exosomal miRNA expression in seven cancer datasets (patients vs. controls).

Cancer	Number of Upregulated miRNAs	Number of Downregulated miRNAs
Prostate cancer	6	25
Gastric cancer	7	-
Colon cancer	39	-
Glioblastoma		21
Multiple myeloma	109	97
Lung cancer	3	10
Liver cancer	19	46

**Table 2:** Identification of common miRNAs (patients vs. controls).

Cancer	Upregulated miRNA (Fold Change)	Downregulated miRNA (Fold Change)
Prostate cancer	miR-543 + (1.16 × 10^−6^)	miR-543 (−5.07), miR-495-3*p* (−4.25)
Gastric cancer	miR-495-3p (1.86), miR-543 (1.47) miR-543 + (1.35 × 10^−10^)	
Colon cancer	miR-495-3p (4.62), miR-543 (5.7) miR-495-3*p* + (3.72 × 10^−12^)	
Glioblastoma		miR-543 (−4.64) miR-495-3*p*–(0.0120)
Multiple myeloma	miR-495-3*p* (1.32)	

**Table 3:** Kyoto Encyclopedia of Genes and Genomes pathways enriched for mRNAs involved in the miR-495-3p community.

Description	Adjusted *p*-Value	Gene ID
GABAergic synapse	0.001	*GABRA1/NSF/GABRA4/GLS/SLC12A5/GAD1/SLC38A2/GAD2/GABBR2/GABRB2/CACNA1D/GABRG1/GABRB3/PRKCB/GABARAP*
AMPK signaling pathway	0.002	*SREBF1/RAB10/EEF2K/ELAVL1/PPP2R2C/PPP2R5C/LIPE/SCD/RHEB/PRKAG2/AKT3/RAB14/PFKFB2/MAP3K7/SCD5/PIK3R1/IRS2*
MAPK signaling pathway	0.020	*HSPA1B/LDLR/TGFBR2/RNF41/RAB10/RAB31/TFRC/EPS15/SNX5/EEA1/RAB11A/RAB5B/ACAP2/SNX4/STAM2/CHMP3/CAPZA1/DNAJC6/RAB22A/HSPA2/PSD3/KIF5B/DNM3/CAPZA2*
Endocytosis	0.0204017	*HSPA1B/LDLR/TGFBR2/RNF41/RAB10/RAB31/TFRC/EPS15/SNX5/EEA1/RAB11A/RAB5B/ACAP2/SNX4/STAM2/CHMP3/CAPZA1/DNAJC6/RAB22A/HSPA2/PSD3/KIF5B/DNM3/CAPZA2*
Oxytocin signaling pathway	0.0204017	*EEF2K/CAMK2A/PTGS2/MEF2C/PRKAG2/ITPR2/CAMK2G/GUCY1A2/CACNA2D1/CACNA1D/RCAN1/CALM3/PRKCB/CAMK1D/ACTG1/CALM1/PPP1CB*

**Table 4:** Summary of miRNA datasets among different cancer types for this study.

Accession Number	Sample Type	Patient Features	Methodological Analysis
GSE130654 [51]	Extracellular vesicles derived from gastric cancer patients	36 non-cardia adenocarcinoma patients (stages I and II) and 12 healthy individuals	DEseq2
GSE111803 [52]	Extracellular vesicles derived from lung cancer patients	5 patients with lung adenocarcinoma and 5 healthy controls	DEseq2
GSE94564 [43]	Extracellular vesicles derived from multiple myeloma patients	10 patients newly diagnosed with MM and 5 healthy individuals	DEseq2
GSE123972 [53]	Extracellular vesicles derived from Hepatocellular carcinoma patients	10 individuals with HCC pooled into 2 libraries and 10 healthy donors pooled into 2 libraries	DEseq2
GSE71008 [54]	Extracellular vesicles derived from colon and prostate cancer patients	100 colon cancer patients and 36 prostate cancer patients, and 50 healthy controls	SAM
GSE122488 [39]	Extracellular vesicles derived from glioblastoma patients	12 patients with glioblastoma, 10 with glioma stages II–III, and 16 healthy controls	SAM

## Data Availability

All data used are publicly available from the URLs included in the paper.

## Supplementary Materials

The following are available online at https://www.mdpi.com/article/10.3390/ncrna8030033/s1: Table S1. DE across seven types of cancer; Figure S1. A Venn diagram was elaborated as a tool to find miRNAs in common between different datasets Figure S2. The complete miRNA–mRNA network based on interaction data from miRNet; Figure S3. Three miRNAs in common to miR-495-3p and miR-543 in KEGG cancer pathways; Figure S4. KEGG metabolic pathways analysis of the target genes; Supplementary file S2. miRNA–mRNA bipartite interaction network.

## Abbreviations

3′ UTR	3′ Untranslated region
AMPK	AMP-activated protein kinase
ceRNA	competitive endogenous RNA
circRNA	Circular RNA
CRC	Colorectal cancer
FDR	False Discovery Rate
GABA	Gamma-aminobutyric acid
GEA	Genomic Expression Archive
GEO	Gene Expression Omnibus
GO	Gene Ontology
KEGG	Kyoto Encyclopedia of Genes and Genomes pathways
lncRNA	Long non-coding RNA
MAPK	Mitogen-activated protein kinase
miR	miRNA
ncRNA	non-coding RNA
RNA	Ribonucleic acid
SNARE	Soluble N-ethylmaleimide-sensitive-factor attachment protein receptor
sRNA	small RNA
