# Supplementary material for: Identification of Two Exosomal miRNAs in Circulating Blood of Cancer Patients by Using Integrative Transcriptome and Network Analysis
Source: Noncoding RNA. 2022 May 12;8(3):33. doi: 10.3390/ncrna8030033 (PMC9149928; doi:10.3390/ncrna8030033)
Supplement: Supplementary file 1 [file ncrna-08-00033-s001.zip › ncrna-1605620-Supplementary/Supplementary file S1_Figures and tables.pdf]

Table S1. DE across seven types of cancer.

Cancer Type	miRNA Upregulated	miRNA Downregulated
Lung	hsa-miR-369-3p,hsa-miR-410-3p,hsa-miR-411-5p	hsa-miR-100-5p, hsa-miR-1246<br>hsa-miR-1255b-5p,<br>hsa-miR-193a-5p, hsa-miR-194-5p<br>hsa-miR-215-5p
Myeloma multiple	hsa-miR-3198, hsa-miR-3198.1<br>hsa-miR-330-3p, hsa-miR-27b-3p<br>hsa-miR-1229-3p, hsa-miR-494<br>hsa-miR-493-3p, hsa-miR-744-5p<br>hsa-miR-93-3p, hsa-miR-539-5p<br>hsa-miR-1185-1-3p, hsa-miR-3179<br>hsa-miR-3179.1,hsa-miR-3179.2<br>hsa-miR-146a-3p,hsa-miR-598<br>hsa-miR-3120-3p,hsa-miR-576-5p<br>hsa-miR-485-5p,hsa-miR-26a-5p.1, hsa-miR-26a-5p,hsa-miR-28-5p<br>hsa-miR-3138,hsa-miR-363-5p<br>hsa-miR-1306-5p,hsa-miR-382-3p<br>hsa-miR-425-3p,hsa-miR-3127-3p<br>hsa-miR-361-3p,hsa-miR-126-5p<br>hsa-miR-154-5p,hsa-miR-135a-3p<br>hsa-miR-301a-3p,hsa-miR-185-3p<br>hsa-miR-495-3p,hsa-miR-374a-5p<br>hsa-miR-215,hsa-miR-3613-3p<br>hsa-miR-409-3p,hsa-miR-192-5p<br>hsa-miR-1908,hsa-miR-19b-3p<br>hsa-miR-19b-3p.1,hsa-miR-3191-3p,hsa-miR-369-5p,hsa-miR-652-3p,hsa-miR-380-5p, hsa-miR-98-3p, hsa-miR-146b-3p<br>hsa-miR-3158-3p,hsa-miR-3158-3p.1,hsa-miR-379-3p,hsa-miR-4435<br>hsa-miR-4435.1,hsa-miR-1301<br>hsa-miR-654-3p,hsa-let-7e-5p<br>hsa-miR-552,hsa-miR-3617-5p<br>hsa-miR-6513-5p,hsa-miR-431-5p<br>hsa-miR-223-3p,hsa-miR-379-5p<br>hsa-miR-2355-5p,hsa-miR-146b-5p,hsa-miR-331-3p,hsa-miR-641<br>hsa-miR-4326,hsa-miR-3135a<br>hsa-miR-339-5p,hsa-miR-889<br>hsa-miR-432-5p,hsa-miR-127-3p<br>hsa-miR-151a-3p,hsa-miR-411-5p	hsa-miR-10b-3p, hsa-miR-222-5p<br>hsa-miR-193b-5p, hsa-miR-1246<br>hsa-miR-10a-3p, hsa-miR-3674<br>hsa-miR-450b-5p, hsa-miR-9-3p<br>hsa-miR-9-3p.1, hsa-miR-9-3p.2<br>hsa-miR-708-3p, hsa-miR-627<br>hsa-miR-1262, hsa-miR-676-3p<br>hsa-miR-642a-3p, hsa-miR-3186-3p<br>hsa-miR-483-5p, hsa-miR-2115-5p<br>hsa-miR-3150b-3p,<br>hsa-miR-132-5p<br>hsa-miR-125b-5p,hsa-miR-125b-5p.1, hsa-miR-5096, hsa-miR-542-3p<br>hsa-miR-145-3p,hsa-miR-424-5p<br>hsa-miR-320c, hsa-miR-501-3p<br>hsa-miR-4755-3p, hsa-miR-3140-3p<br>hsa-miR-551a, hsa-miR-2115-3p<br>hsa-miR-6503-3p, hsa-miR-576-3p<br>hsa-miR-33b-3p, hsa-miR-338-5p<br>hsa-miR-3186-5p, hsa-miR-502-3p

	hsa-miR-1271-5p,hsa-miR-5010-3p,hsa-miR-877-5p,hsa-miR-374b-5p,hsa-miR-199a-5p<br>hsa-miR-199a-5p.1,hsa-miR-1249<br>hsa-miR-411-3p,hsa-miR-150-5p<br>hsa-miR-337-3p,hsa-miR-381-3p<br>hsa-miR-766-5p,hsa-miR-1185-2-3p,hsa-miR-758-3p<br>hsa-miR-409-5p,hsa-miR-329<br>hsa-miR-329.1,hsa-miR-487b<br>hsa-miR-370,hsa-miR-574-3p<br>hsa-miR-376a-3p,hsa-miR-376a-3p.1,hsa-miR-3200-5p<br>hsa-miR-654-5p,hsa-miR-324-5p<br>hsa-miR-376c-3p,hsa-miR-483-3p<br>hsa-miR-6721-5p,hsa-miR-23b-3p<br>hsa-miR-326,hsa-miR-5193<br>hsa-miR-3653,hsa-miR-766-3p<br>hsa-miR-624-5p,hsa-miR-664a-3p<br>hsa-miR-133a,hsa-miR-133a.1<br>hsa-miR-939-5p,hsa-miR-30b-5p<br>hsa-miR-4446-3p,hsa-miR-505-5p<br>hsa-miR-1296,hsa-miR-133b<br>hsa-miR-296-5p,hsa-miR-122-3p<br>hsa-miR-3609	hsa-miR-320b, hsa-miR-4482-3p<br>hsa-miR-335-5p, hsa-miR-4755-5p<br>hsa-miR-4440, hsa-miR-450a-3p<br>hsa-miR-1284, hsa-miR-1299<br>hsa-miR-129-5p, hsa-miR-129-5p.1<br>hsa-miR-378i, hsa-miR-378c<br>hsa-miR-99a-5p, hsa-miR-22-5p<br>hsa-miR-21-3p, hsa-miR-10b-5p<br>hsa-let-7d-3p, hsa-miR-378a-3p<br>hsa-miR-873-3p, hsa-miR-500a-3p<br>hsa-miR-3187-3p, hsa-miR-3675-5p<br>hsa-miR-548l,hsa-miR-3918<br>hsa-miR-4443, hsa-miR-181b-5p<br>hsa-miR-181b-5p.1,<br>hsa-miR-218-5p.1, hsa-miR-218-5p<br>hsa-miR-1250, hsa-miR-378f<br>hsa-miR-422a, hsa-miR-378d.1<br>hsa-miR-6509-5p, hsa-miR-4470<br>hsa-miR-5588-5p, hsa-miR-452-5p<br>hsa-miR-125a-3p, hsa-miR-193a-5p<br>hsa-miR-188-5p, hsa-miR-27a-5p<br>hsa-miR-130b-3p, hsa-miR-629-5p<br>hsa-miR-499a-5p, hsa-miR-320b.1<br>hsa-miR-3615, hsa-miR-618<br>hsa-miR-100-5p, hsa-miR-3605-3p

Hepatocarcinoma	hsa-mir-4435-2 hsa-mir-4435-1<br>hsa-mir-3191,hsa-mir-6852<br>hsa-mir-4446,hsa-mir-493<br>hsa-mir-628,hsa-mir-6721<br>hsa-mir-3120,hsa-mir-654<br>hsa-mir-5584,hsa-mir-584<br>hsa-mir-380,hsa-mir-381<br>hsa-mir-1273h,hsa-mir-5187<br>hsa-mir-151a,hsa-mir-432<br>hsa-mir-379,hsa-mir-1271<br>hsa-mir-4659b	hsa-mir-320d-1,hsa-mir-144<br>hsa-mir-422a,hsa-mir-125b-2<br>hsa-mir-320e,hsa-mir-4508<br>hsa-mir-378a,hsa-mir-193b<br>hsa-mir-320d-2,hsa-mir-193a<br>AC021590.1,hsa-mir-378g<br>AC099677.4,hsa-mir-671<br>hsa-mir-6503,hsa-mir-1228<br>hsa-mir-8086,hsa-mir-378i<br>hsa-mir-3664,AC016601.1<br>hsa-mir-378b,hsa-mir-885<br>hsa-mir-362,hsa-mir-100<br>AC098818.3,hsa-mir-3960<br>hsa-mir-1180,hsa-mir-378d-2<br>hsa-mir-378d-1,hsa-mir-1246<br>hsa-mir-192,hsa-mir-455<br>hsa-mir-3611,hsa-mir-3929<br>hsa-mir-122,hsa-mir-122b<br>AL031428.2,hsa-mir-214<br>AL928646.1,hsa-mir-483<br>hsa-mir-378c,hsa-mir-7704<br>AC104450.1,hsa-mir-4488<br>hsa-mir-217,hsa-mir-675<br>hsa-mir-216a
Gastric cancer	hsa-miR-105-5p, hsa-miR-495-3p	

	hsa-miR-129-5p, hsa-miR-543<br>hsa-miR-382-5p, hsa-miR-218-5p<br>hsa-miR-889-3p	
GBM		hsa-miR-21-5p,hsa-miR-671-3p<br>hsa-miR-369-5p, hsa-miR-191-3p<br>hsa-miR-454-5p,hsa-miR-4659b-3p<br>hsa-miR-543,hsa-miR-331-3p<br>hsa-miR-1260b,hsa-miR-98-3p<br>hsa-miR-339-5p,hsa-miR-26b-3p<br>hsa-miR-301a-5p,hsa-miR-199a-5p<br>hsa-miR-744-3p,hsa-miR-340-5p<br>hsa-miR-340-3p,hsa-miR-26a-5p<br>hsa-miR-328-3p,hsa-miR-485-3p<br>hsa-miR-128-3p
CCR	hsa-miR-1343-3p,hsa-miR-125a-5p,hsa-miR-381-3p,hsa-miR-128-3p,hsa-miR-139-5p,hsa-miR-92b-3p,hsa-miR-495-3p,hsa-miR-212-5p,hsa-miR-132-3p,hsa-miR-885-5p,mmu-miR-872-3p,rno-miR-872-3p,hsa-miR-30e-3p,hsa-miR-708-5p,mmu-miR-872-5p,rno-miR-872-5p,eca-miR-872,cgr-miR-<br>hsa-miR-143-3p,hsa-miR-140-5p<br>hsa-miR-30a-3p,hsa-miR-10b-5p<br>hsa-miR-218-5p,chi-let-7b-3p<br>hsa-miR-28-3p,hsa-miR-195-5p<br>hsa-miR-409-3p,hsa-miR-7-5p<br>hsa-miR-206,hsa-let-7c-5p<br>hsa-miR-29a-3p,hsa-miR-126-5p<br>hsa-miR-129-1-3p,hsa-miR-137<br>hsa-miR-340-5p,hsa-miR-27b-3p<br>hsa-miR-411-5p,hsa-miR-889-3p<br>hsa-miR-9-5p,hsa-miR-148b-3p<br>hsa-miR-543,hsa-miR-204-5p<br>hsa-let-7g-5p	

Prostate	hsa-miR-204-3p, hsa-let-7i-3p<br>hsa-miR-423-5p, hsa-miR-490-5p<br>hsa-miR-130a-3p, hsa-miR-125a-3p	hsa-miR-92b-3p, hsa-miR-26b-5p<br>hsa-miR-93-5p, hsa-miR-30c-5p<br>hsa-miR-20a-5p, hsa-let-7f-5p<br>hsa-miR-885-5p, hsa-miR-126-3p<br>hsa-miR-495-3p, hsa-miR-139-5p<br>hsa-miR-26a-5p, hsa-miR-132-3p<br>hsa-miR-381-3p, hsa-miR-212-5p<br>hsa-let-7a-5p, hsa-miR-191-5p<br>hsa-miR-30e-3p, hsa-let-7d-5p<br>hsa-let-7e-5p, hsa-miR-128-3p<br>hsa-miR-543, hsa-miR-7-5p<br>hsa-miR-204-5p, hsa-miR-125a-5p<br>hsa-miR-1343-3p

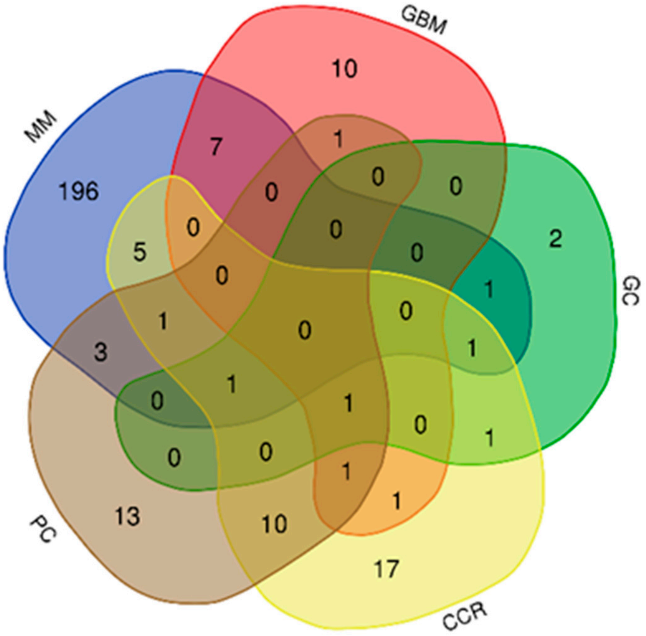

Figure S1. *As a tool to find miRNAs in common between the different datasets, a Venn diagram was elaborated.*

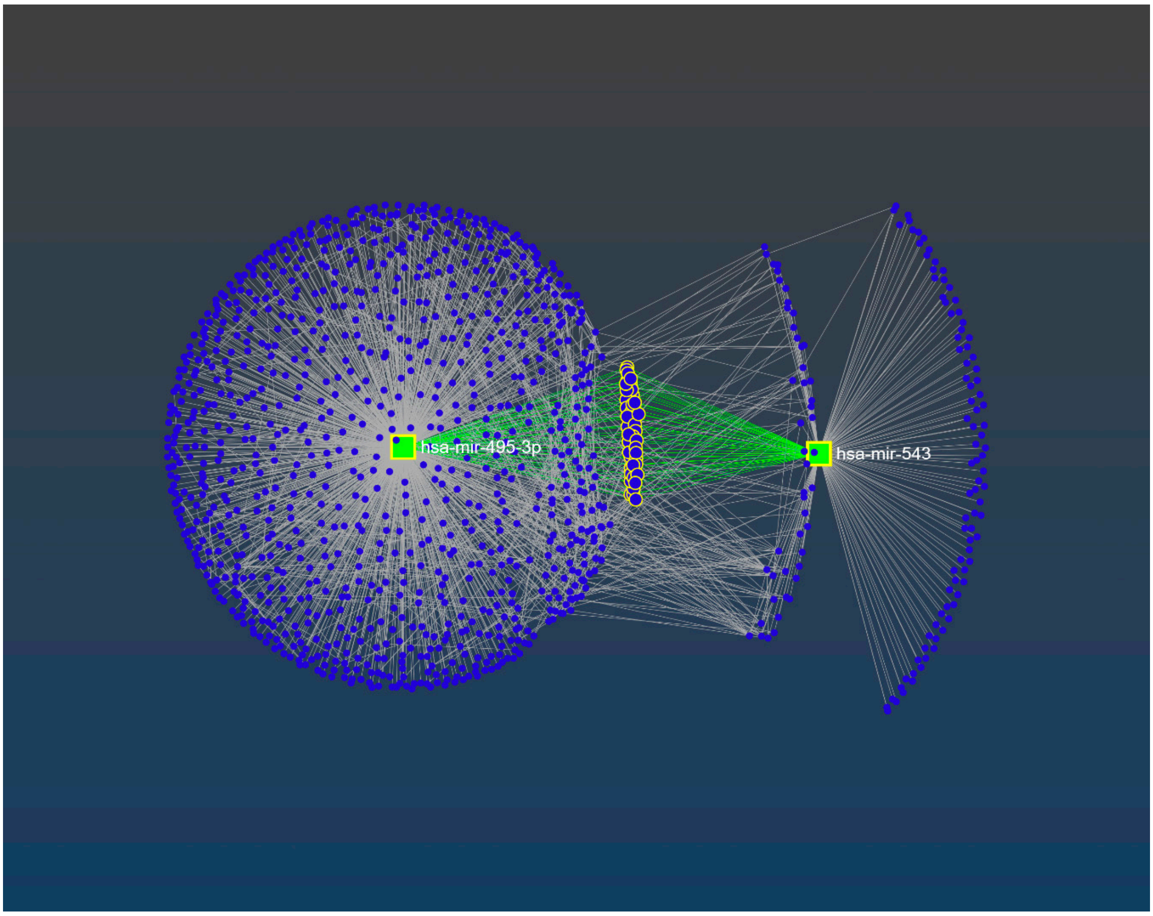

Figure S2. *The complete miRNA-mRNA network based on the interaction data from miRNet. Square indicates miRNAs, and the blue circle indicates mRNA predicted target.*

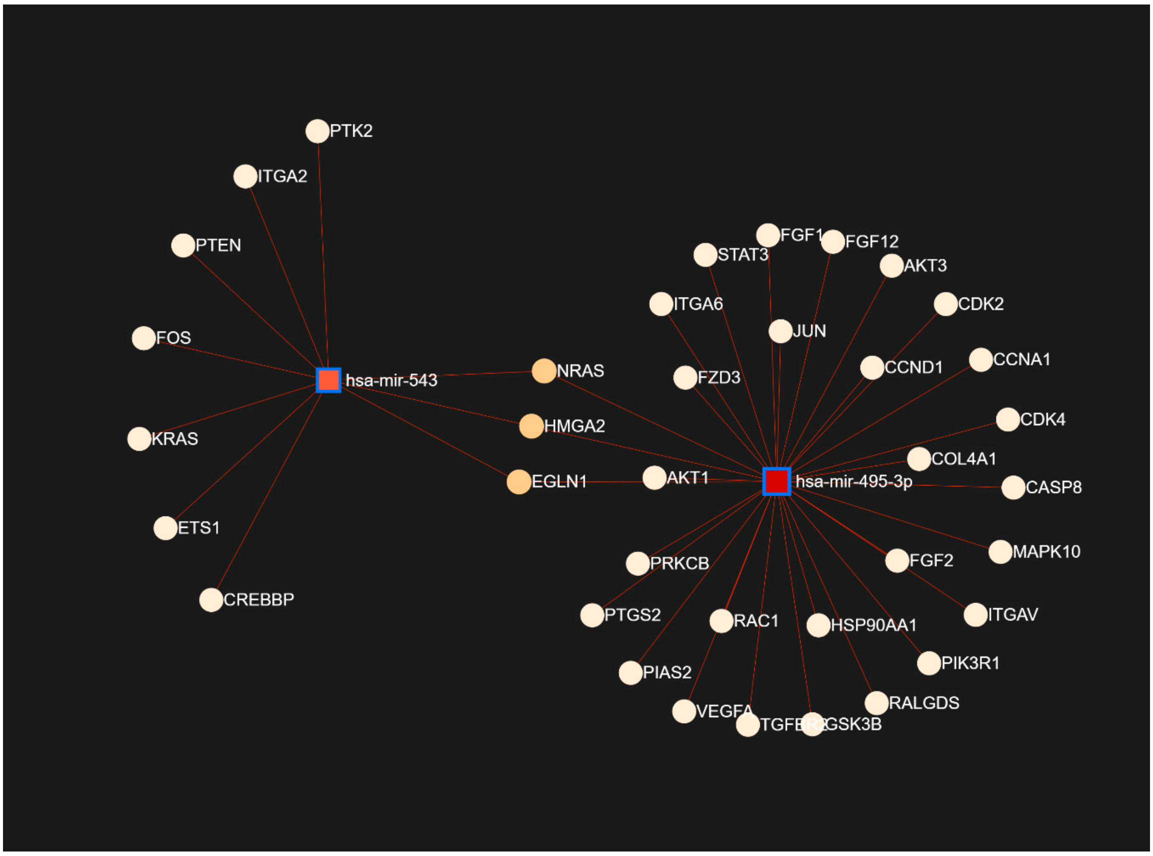

Figure S3. *Three miRNAs in common into miR-495-3p and miR-543 in cancer pathways of KEGG.*

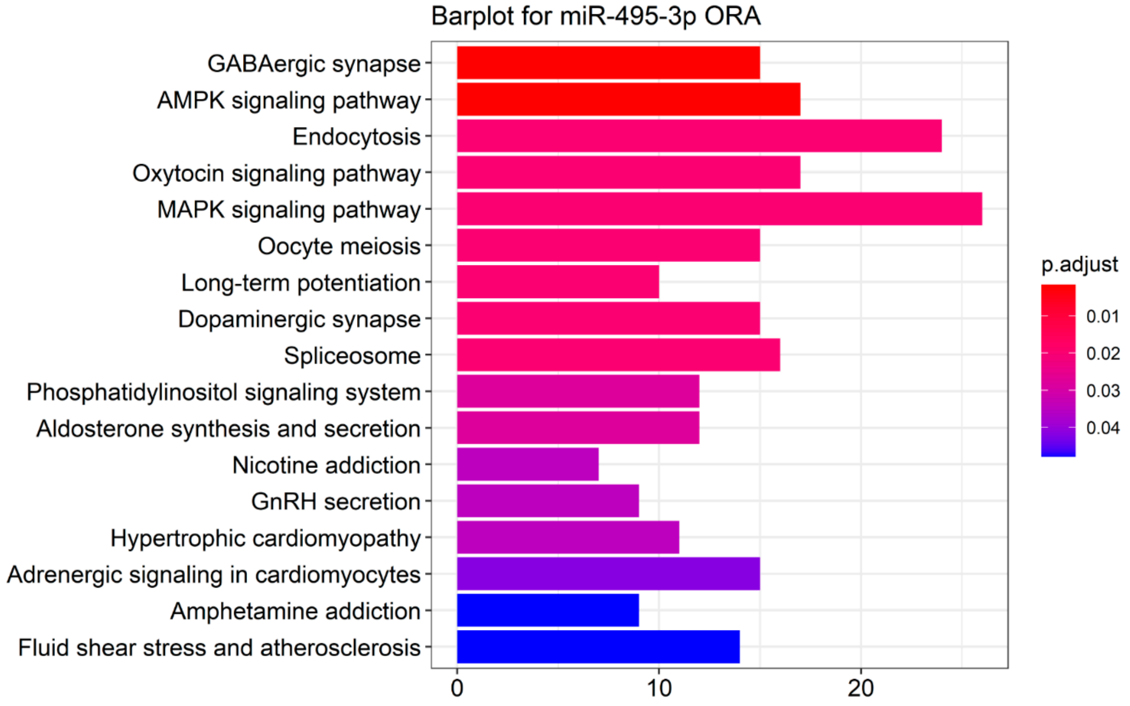

Figure S4. *KEGG metabolic pathways analysis of the target genes. KEGG pathways enriched by DE miRNA are involved in the biggest miRNA-mRNA community.*
